# Metabolite-Related Evidence Linking Metabolic Measures to Patient-Reported Outcomes in Inflammatory Bowel Disease: A Critical Narrative Review

**DOI:** 10.3390/metabo16090696

**Published:** 2026-09-20

**Authors:** Patrycja Krynicka, Aleksandra Helena Piotrowska, Maria Kłopocka

**Affiliations:** Department of Gastroenterology and Nutrition Disorders, Collegium Medicum in Bydgoszcz, Nicolaus Copernicus University, 85-168 Bydgoszcz, Poland; aleksandra.piotrowska18@gmail.com (A.H.P.);

**Keywords:** inflammatory bowel disease, metabolomics, metabolites, microbiota-derived metabolites, patient-reported outcomes, fatigue, bile acids, short-chain fatty acids, tryptophan metabolism, critical narrative review

## Abstract

Background/Objectives: Patient-reported outcomes (PROs) capture important dimensions of inflammatory bowel disease (IBD), including fatigue, questionnaire-defined mood-related symptom burden, pain, sleep disturbance, bowel urgency and impaired health-related quality of life, yet these outcomes are incompletely explained by conventional inflammatory markers. Metabolite-related measures may capture host–microbe co-metabolism relevant to patient-reported burden. Methods: This critical narrative review synthesized adult IBD literature linking metabolite-related measures with patient-reported symptom domains. PubMed/MEDLINE, Embase.com, Scopus and Web of Science Core Collection were searched through 11 June 2026; purposive source selection supported critical narrative synthesis rather than exhaustive enumeration. Results: Our structured search identified only a small number of human studies, mainly in fatigue and questionnaire-defined mood-related symptom burden. Studies included serum, plasma or fecal metabolite measurements, exploratory multi-omics, inferred microbial metabolic functions and interventional pathway testing. Fatigue was associated with serum amino-acid-related changes, plasma lipidomic alterations and fecal untargeted metabolomic and microbiome differences; inferred microbial functional modules were also reported. Mood-related burden was associated with serum and fecal metabolomic changes, bile-acid profiles and microbiota-derived functional signals. Within our search and eligibility criteria, no human studies paired measured metabolites with sleep-, pain- or urgency-specific patient-reported outcome measures (PROMs); evidence for multidimensional clusters was insufficient. Conclusions: We did not identify a metabolite-related signature for the assessment of patient-reported burden that had undergone both analytical and clinical validation; findings support candidate associations and exploratory signatures. Future work requires longitudinal, phenotype-stratified cohorts, validated PROMs, rigorous metabolomics methodology, causal modeling, inflammatory and dietary metadata, multiple-testing control and independent validation for clinical translation.

## 1. Introduction

Inflammatory bowel disease (IBD), including Crohn’s disease (CD) and ulcerative colitis (UC), is increasingly managed using treat-to-target frameworks that extend beyond symptom relief toward objective control of inflammation, mucosal healing and prevention of structural damage [1]. Novel outcome frameworks emphasize that patient-reported domains are clinically important in IBD [2]. Core-outcome work has similarly highlighted the need to capture outcomes meaningful to patients [3]. Patient-reported outcome measures may correlate poorly with objective inflammatory bowel disease activity measures [4]. Real-world IBD-PODCAST data show that suboptimal disease control is associated with persistent patient burden [5]. Canadian IBD-PODCAST data further link suboptimal disease control with reduced quality of life [6].

A patient-reported outcome (PRO) is the concept reported directly by the patient, such as fatigue, pain, urgency, sleep disturbance or perceived quality of life. A patient-reported outcome measure (PROM) is the instrument used to measure that concept. This distinction is important because symptom-specific PROs, psychological symptom burden, health-related quality of life (HRQoL), multidimensional symptom burden and formal psychiatric diagnoses are not interchangeable. Throughout this review, depression is distinguished from depressive symptoms, anxiety disorder from anxiety symptoms, psychological disorder from psychological burden, and screening-scale positivity from clinical diagnosis.

Common instruments discussed in this review include the Functional Assessment of Chronic Illness Therapy-Fatigue scale (FACIT-F), Fatigue Scale-14 (FS-14), Patient-Reported Outcomes Measurement Information System Fatigue instruments (PROMIS-Fatigue), Patient Health Questionnaire-9 (PHQ-9), Generalized Anxiety Disorder 7-item scale (GAD-7), Hospital Anxiety and Depression Scale (HADS), Wuerzburger Fatigue Inventory for Multiple Sclerosis (WEIMuS) and fatigue visual analogue scale (fVAS). These instruments may be analyzed as continuous scores or dichotomized using cut-off values; dichotomization can improve clinical interpretability but may reduce information and should be interpreted cautiously.

PROs should therefore not be treated as patient-reported proxies for objective disease-activity measures. Rather, they measure dimensions of disease burden that conventional biomarkers cannot directly capture. A patient with normalized fecal calprotectin and improved endoscopic activity may still experience fatigue, altered bowel confidence, cognitive burden or mood-related symptoms. Conversely, active mucosal inflammation may occur with relatively few symptoms. This bidirectional mismatch creates a need for frameworks that integrate, rather than replace, objective inflammatory and patient-reported dimensions.

The gut-brain axis provides a biologically coherent framework for interpreting persistent burden in IBD. Bidirectional brain-gut effects influence mood and prognosis in IBD [7]. IBD-related fatigue has been discussed in relation to inflammatory, psychological, nutritional and gut-brain-axis mechanisms [8]. Broader gut-brain frameworks also include neural, immune, sleep, stress-response and behavioral pathways [9]. Microbial metabolites are a key component of microbiota-gut-brain signaling in IBD [10]. Within this network, metabolomics is particularly attractive because metabolites represent functional outputs of host-microbe co-metabolism and may be closer to clinical phenotypes than taxonomy alone. Large IBD multi-omics work supports joint consideration of microbial, metabolic and host-response layers [11]. Integrative multi-omics approaches are increasingly used to study host-microbiome interactions in IBD [12]. Recent host-microbiota interactome analyses further support combining microbial, metabolic and host-response data [13].

Short-chain fatty acids (SCFAs), bile acids, tryptophan–kynurenine metabolites, serotonin-related metabolites, microbial indoles, lipid mediators, volatile organic compounds (VOCs) and broader untargeted metabolomic signatures are biologically plausible contributors to patient-reported burden. However, pathway plausibility is not equivalent to clinical validity. Many IBD metabolomic studies focus on diagnosis, inflammatory activity, treatment response or mechanistic discovery rather than validated PROMs. Conversely, many PRO studies do not include metabolomics or functional microbial readouts. A critical narrative synthesis is therefore needed to distinguish candidate metabolite–PRO associations from validated clinical tools.

In this review, a metabolite-related measure refers to a directly quantified metabolite, metabolite class or metabolomic feature measured in a biological matrix, or to a metabolite-associated microbial functional pathway when the distinction from direct metabolite quantification is explicitly stated. A metabolic signature refers to a pattern of measured metabolites or annotated metabolic features associated with a patient-reported domain, not a clinically validated biomarker unless independent validation and context-of-use evidence are available. Predicted microbial functions, metabolite-associated taxa and pathway-enrichment analyses without direct metabolite measurement were treated as evidence of potential metabolic capacity or biological context, not measured metabolite concentrations, pathway activity or metabolic flux. Interventional studies such as 5-hydroxytryptophan supplementation are interpreted as tests of a specific metabolic-pathway hypothesis, not as studies identifying or validating metabolic signatures.

Previous reviews have summarized different parts of this field. Fatigue-focused reviews have discussed gut-brain-axis mechanisms and symptom burden in IBD [8]. Systematic metabolomics reviews have synthesized IBD metabolic alterations mainly in relation to diagnosis, disease activity and pathophysiology [14]. Broader metabolomics reviews have addressed IBD pathogenesis, prognosis and treatment response [15]. Recent microbiota-derived metabolite reviews have emphasized therapeutic and mechanistic targets in IBD [16]. Zarei et al. recently reviewed gut-microbiota-derived metabolomic profiles in relation to mental disorders, particularly anxiety and depression, in IBD [17]. The most recent broad systematic review evaluated metabolomics for IBD diagnosis, disease differentiation, activity assessment, treatment response and monitoring, rather than organizing the evidence around PROs or PROMs [18]. The present review extends beyond mood-related symptom burden to fatigue, sleep disturbance, pain, bowel urgency, stool-frequency burden and multidimensional symptom clusters; uses PROs and PROMs as its organizing framework; distinguishes directly measured metabolites at the level of individual studies from exploratory multi-omics signals, inferred microbial metabolic functions and interventional pathway evidence; and does not treat screening-scale positivity as equivalent to a formal psychiatric diagnosis. A comparison of the scope and methodological focus of previous reviews and the present review is provided in Supplementary Table S1.

The objective of this critical narrative review was therefore to synthesize and critically interpret literature on metabolite-related measures and metabolic signals associated with PROs in IBD, while explicitly separating the relevant evidence layers and identifying methodological constraints that currently limit clinical translation.

## 2. Approach to Literature Identification and Narrative Synthesis

This article was designed as a critical narrative review aimed at synthesizing adult human and mechanistic literature relevant to metabolite-related measures and PROs in IBD. Source identification was purposive and intended to support comprehensive critical interpretation. Publications were considered according to their relevance to the review question, methodological informativeness, biological plausibility and contribution to the conceptual interpretation of metabolite-PRO relationships.

PubMed/MEDLINE, Embase.com, Scopus, and Web of Science Core Collection were searched from database inception to 11 June 2026. Search strategies combined three concept blocks covering: (1) inflammatory bowel disease; (2) metabolomics, directly measured metabolites, microbiota-derived metabolites, metabolic pathways, and inferred microbial metabolic functions; and (3) PROs, PROMs, health-related quality of life, and relevant patient-reported symptom domains. The complete database-specific search strategies are provided in Supplementary Table S2. No publication-date, language, age, species, or study-design filters were applied at the search stage. Reference lists of relevant reviews and key primary publications were additionally screened manually. Across the four databases, 827 records were identified, and manual citation searching yielded 32 additional records. After removal of 312 duplicates, 547 unique records underwent title/abstract screening; 44 full-text reports were assessed, and eight studies met the operational criteria for inclusion in the principal human evidence synthesis.

The review prioritized adult human publications in Crohn’s disease, ulcerative colitis or IBD-unclassified that evaluated directly measured metabolites, metabolomic signatures, metabolite-associated microbial functional pathways or other metabolite-related measures in relation to PROs, PROMs or clearly defined patient-reported symptom domains. The literature identification and narrative review were performed by A.H.P. and P.K.; decisions regarding which publications were central to the principal human discussion were made through author discussion, with emphasis on direct relevance, availability of patient-reported outcomes in the same clinical context and methodological contribution to the narrative synthesis.

Evidence not prioritized as part of the principal human discussion comprised pediatric-only literature, animal-only or in vitro work, taxonomy-only microbiome studies without functional or metabolite-level inference, metabolomic studies without an interpretable patient-reported outcome, studies focused exclusively on objective inflammatory activity, conference abstracts lacking sufficient methodological detail and reports with insufficient methodological information. Such sources were used only as mechanistic or contextual evidence when they helped interpret biological plausibility or research priorities. At the full-text eligibility stage, the review was restricted to peer-reviewed publications available in English; no translation workflow for non-English full texts was undertaken.

For narrative organization, human publications were grouped into four categories: evidence based on direct metabolite measurement, exploratory multi-omics evidence, evidence from inferred microbial metabolic functions and interventional pathway evidence. Evidence based on direct metabolite measurement refers to studies in which metabolites were directly quantified in biological material and analyzed in relation to PROs. Exploratory multi-omics evidence refers to studies in which metabolomics was integrated with microbiome, proteomic or other omics layers. Evidence from inferred microbial metabolic functions refers to studies in which functions or modules were predicted from metagenomic or microbiome data without direct metabolite quantification. Interventional pathway evidence refers to studies in which a defined metabolic pathway was modified and both metabolic response and PROs were evaluated.

This categorization was used only to organize the narrative synthesis. It does not represent a formal quality-grading system, a certainty-of-evidence assessment or a hierarchy of clinical validity. In particular, “direct” indicates only that metabolites were measured in the biological material; it does not imply stronger evidence, lower risk of bias or greater certainty. Studies with directly measured metabolites may still be small, cross-sectional, exploratory, affected by confounding or lacking independent validation. Mechanistic studies, animal studies, in vitro studies, taxonomy-only microbiome studies and studies that did not assess metabolite-related measures and PROs in the same clinical context are referred to as mechanistic or contextual evidence and are used in interpretative sections rather than being treated as equivalent to the principal human literature summarized in Table 1. Statements concerning evidence gaps were bounded to the scope of the structured search; failure to identify a study or validation was not interpreted as proof of universal absence.

The coherence and transparency of the review were considered using principles consistent with the Scale for the Assessment of Narrative Review Articles (SANRA), including justification of the review’s importance, statement of aims, description of literature identification, appropriate referencing, scientific reasoning and balanced presentation of evidence [19]. Critical methodological appraisal was descriptive and focused on study design, sample size, IBD phenotype and activity, PROM adequacy, synchronization of biospecimen and PROM assessment, inflammatory and dietary context, metabolomics quality, multiple-testing control, risk of overfitting and internal or external validation. The characteristics, principal findings, evidence classification, and integrated critical methodological appraisal of the key human studies are presented in Table 1.

**Table 1:** Key Human Studies Linking Metabolite-Related Measures with Patient-Reported Outcomes in Inflammatory Bowel Disease.

Study and Design	Population and IBD Phenotype/Activity	PRO/PROM	Biological Matrix and Metabolite-Related Assessment	Main Findings	Evidence Category	Major Confounders Measured or Statistically Considered; Key Limitations
**Borren et al., 2021** [20] Prospective cohort; cross-sectional multi-omics comparison.	166 adults with clinically and endoscopically quiescent IBD: 106 CD and 60 UC. Omics subsets: serum metabolomics, *n* = 156; serum proteomics, *n* = 131; fecal microbiome, *n* = 50.	FACIT-F; prespecified fatigue threshold and continuous-score analyses.	Targeted serum metabolomics integrated with serum proteomics and fecal microbiome profiling.	An 18-metabolite serum cluster differed between fatigued and non-fatigued participants. Methionine, tryptophan, proline, and sarcosine were lower in fatigued participants. Fatigue was also associated with depletion of butyrate-associated taxa, including *Faecalibacterium prausnitzii* and *Roseburia hominis*.	Exploratory multi-omics evidence including directly measured serum metabolites.	Measured/considered: clinical and endoscopic remission; adjusted models included age, sex, body mass index, smoking and IBD type. Key limitations: cross-sectional symptom comparison; unequal omics subsets; no control for diet, anemia/iron status, sleep or physical activity; limited metabolite panel; no measured short-chain fatty acids or metabolic flux; multiplicity concerns; no external validation.
**Horta et al., 2021** [21] Exploratory cross-sectional plasma lipidomics study.	47 adults with quiescent IBD: 27 CD and 20 UC; 23 fatigued and 24 non-fatigued.	FACIT-FS; participants grouped as fatigued versus non-fatigued.	Direct plasma lipidomic profiling using UPLC-TOFMS, with multivariate, network and pathway analyses.	Fatigue was associated with lower phosphatidylcholines, plasmanyls, sphingomyelins, lysophosphatidylcholines, phosphatidylethanolamines, phosphatidylinositols, phosphatidylserines and eicosanoids. Arachidonic-acid and glycerophospholipid metabolism and the sphingolipid pathway were dysregulated.	Evidence based on directly measured plasma lipids.	Measured/considered: IBD type and clinical activity, sex, age, body mass index, smoking, illness duration and interleukins 5, 8 and 12. Key limitations: small cross-sectional sample; dichotomized fatigue; high-dimensional selection and annotation uncertainty; modest cross-validation; no covariate-adjusted metabolite model or independent validation.
**Liu et al., 2026** [22] Cross-sectional four-group observational multi-omics study.	120 adults: UC with fatigue, *n* = 30; UC without fatigue, *n* = 30; healthy participants with fatigue, *n* = 30; healthy participants without fatigue, *n* = 30. UC groups included active and quiescent disease; active or severe disease was numerically more frequent in the fatigue group.	FS-14; fatigue defined as FS-14 ≥ 7. A traditional Chinese medicine fatigue score was also recorded.	Direct untargeted fecal LC–MS/MS integrated with fecal 16S rRNA profiling.	UC with fatigue versus UC without fatigue showed 262 differential fecal metabolites. Linoleoyl ethanolamide differed between groups, arachidonoyl ethanolamide and glycocholic acid were increased, and thromboxane was detected only in the UC-with-fatigue group; eicosanoid, tryptophan and tyrosine pathways were implicated.	Exploratory multi-omics evidence including directly measured fecal metabolites.	Measured/considered: age, sex, body mass index, Mayo score, disease phase and extent; recent antibiotic/probiotic use was excluded. Key limitations: no multivariable adjustment; possible residual confounding by activity/severity, diet, medication, transit and stool water; high-dimensional selection and annotation uncertainty; unclear multiplicity control; no independent validation.
**Thomann et al., 2022** [23] Prospective observational study using fecal metagenomics and Bayesian modeling.	62 adults with active IBD, predominantly CD; *n* = 57 in the final network analysis.	HADS-D for depressive symptom severity; WEIMuS for fatigue.	Fecal metagenomic profiling with KEGG-based functional modules and Bayesian network modeling; metabolites were not directly measured.	Depressive symptom severity and fatigue were associated with microbial taxa and predicted functional modules related to amino-acid metabolism, methionine pathways, central carbohydrate metabolism, pentose-phosphate pathways, glycan or pectin degradation, and SCFA-associated taxa.	Evidence from inferred microbial metabolic functions; no metabolites were measured.	Measured/considered: age, sex, C-reactive protein, fecal calprotectin, clinical activity and treatment; abundance data were adjusted for age, sex, C-reactive protein, steroids, mesalamine, immunosuppressants, hormonal contraceptives, antidepressants and proton-pump inhibitors. Key limitations: active-disease cohort, predominantly Crohn’s disease; inferred metabolic capacity rather than measured concentrations, activity or flux; no external validation.
**Yuan et al., 2021** [24] Prospective observational multi-omics study with mechanistic animal experiments.	129 patients with active UC enrolled in two phases (Phase 1, *n* = 69; Phase 2, *n* = 60), 49 non-IBD participants with depressive or anxiety symptoms, and 62 healthy controls; total *n* = 240.	PHQ-9 and GAD-7; threshold-defined depressive and anxiety symptoms.	Fecal 16S rRNA profiling was performed across the broader study population. Serum LC–MS metabolomics and proteomics were restricted to Phase 2 (*n* = 60; proteomics used pooled samples); selected metabolites were additionally investigated in mice.	Active UC with mood-related symptoms was associated with reduced microbial richness and diversity and altered serum metabolomic and proteomic networks. Glycochenodeoxycholate and several other metabolites were increased, whereas 2′-deoxy-D-ribose and L-pipecolic acid were decreased.	Exploratory multi-omics evidence including directly measured serum metabolites.	Measured/considered: age, sex, body mass index and contemporaneous Mayo/endoscopic activity; recent mood-directed medication was absent. Key limitations: regression covariates were not reported in the main article; active ulcerative colitis only; possible residual inflammatory, dietary, medication and behavioral confounding; high-dimensional analyses, pooled proteomics and no external validation.
**Feng et al., 2022** [25] Cross-sectional clinical study.	39 patients with CD and 14 healthy controls.	Zung Self-Rating Anxiety Scale (SAS) and Zung Self-Rating Depression Scale (SDS); psychological-symptom subgroup defined as SAS ≥ 45 and SDS ≥ 50.	Direct fecal and serum bile-acid profiling using LC–MS/MS, combined with gut microbiome profiling.	Psychological symptom burden in CD was associated with fecal and serum bile-acid alterations and gut microbiota dysbiosis. Correlations involved primary/secondary and conjugated/unconjugated species.	Evidence based on directly measured fecal and serum bile acids.	Measured/considered: age, sex, body mass index, Crohn’s Disease Activity Index and Montreal phenotype; liver/biliary disease and recent antibiotics/cathartics were excluded. Key limitations: small cross-sectional sample; no multivariable adjustment; possible effects of ileal phenotype/resection, diet, medication, transit and stool water; no independent validation.
**Wang et al., 2025** [26] Observational microbiome–metabolomics study.	59 adults with IBD: 32 in remission and 27 with active disease; both CD and UC were included.	HADS; HADS-A ≥ 8 for anxiety symptoms and HADS-D ≥ 8 for depressive symptoms.	Fecal 16S rRNA profiling, Tax4Fun2 functional prediction, and untargeted LC–MS metabolomics with KEGG, HMDB, and LIPID MAPS annotation.	In the anxiety-symptom group, 3-methyl-2-oxobutanoic acid was upregulated, alongside enrichment of predicted valine, leucine and isoleucine degradation. In the depressive-symptom group, α-ketoglutaric acid, pipecolic acid and glycocholic acid were downregulated; predicted butanoate metabolism and lysine degradation were enriched, whereas primary bile-acid biosynthesis was depleted. In the combined anxiety- and depressive-symptom group, α-ketoglutaric acid, taurochenodeoxycholic acid and 2-keto-4-methylthiobutyric acid were downregulated, whereas N6,N6,N6-trimethyl-L-lysine and indole-3-acetic acid were upregulated.	Evidence based on directly measured fecal metabolites, with inferred microbial functional context.	Measured/considered: age, sex, body mass index, IBD type/course/activity, treatment, smoking and alcohol; recent systemic steroids, psychotropics and antibiotics were excluded; beta-diversity models adjusted for age, sex, activity and body mass index. Key limitations: metabolite comparisons were not clearly covariate-adjusted; small cross-sectional cohort; predicted microbial functions; annotation and multiplicity uncertainty; no external validation.
**Truyens et al., 2022** [27] Multicenter randomized placebo-controlled crossover trial.	166 adults with quiescent IBD and fatigue, predominantly CD.	fVAS; primary endpoint defined as a reduction of at least 20%. Secondary outcomes included mood- and stress-related PROs.	Targeted serum assessment of the tryptophan–serotonin pathway during oral 5-HTP supplementation.	Oral 5-HTP increased serum 5-HTP and serotonin concentrations but did not improve fatigue or mood- and stress-related PROs compared with placebo.	Interventional pathway evidence.	Controlled by randomized crossover design; mixed models included intervention, period, intervention-by-period interaction and participant; remission and stable therapy were required, and anemia, major nutrient deficiencies, hypothyroidism and psychiatric comorbidity were excluded. Key limitations: single-pathway intervention; peripheral changes did not establish central target engagement; replication in phenotyped subgroups is needed.

Category note: Evidence categories describe the nature of the evidence, not methodological quality or certainty; “direct” denotes measured metabolites only. Predicted microbial functions, metabolite-associated taxa and pathway-enrichment analyses indicate potential metabolic capacity or biological context, not metabolite concentrations, pathway activity or metabolic flux. Bold text indicates individual study citations. Abbreviations: 5-HTP, 5-hydroxytryptophan; 16S rRNA, 16S ribosomal RNA; CD, Crohn’s disease; FACIT-F, Functional Assessment of Chronic Illness Therapy–Fatigue; FACIT-FS, Functional Assessment of Chronic Illness Therapy–Fatigue subscale; fVAS, fatigue visual analogue scale; FS-14, Fatigue Scale-14; GAD-7, Generalized Anxiety Disorder 7-item scale; HADS, Hospital Anxiety and Depression Scale; HADS-A, Hospital Anxiety and Depression Scale–Anxiety; HADS-D, Hospital Anxiety and Depression Scale–Depression; HMDB, Human Metabolome Database; IBD, inflammatory bowel disease; KEGG, Kyoto Encyclopedia of Genes and Genomes; LC–MS, liquid chromatography–mass spectrometry; LC–MS/MS, liquid chromatography–tandem mass spectrometry; PHQ-9, Patient Health Questionnaire-9; PRO, patient-reported outcome; SAS, Zung Self-Rating Anxiety Scale; SDS, Zung Self-Rating Depression Scale; SCFA, short-chain fatty acid; UC, ulcerative colitis; UPLC-TOFMS, ultra-performance liquid chromatography–time-of-flight mass spectrometry; WEIMuS, Wuerzburger Fatigue Inventory for Multiple Sclerosis.

## 3. Current Human Evidence Linking Metabolite-Related Measures to Patient-Reported Outcomes

### 3.1. Overview of the Current Evidence Base

Our structured search identified only a small number of human studies directly evaluating associations between metabolite-related measures and PROs in IBD, and the identified studies were methodologically heterogeneous. The identified studies were concentrated primarily in fatigue and questionnaire-defined mood-related symptom burden. Within the limits of the databases searched, the search strategy and the operational eligibility criteria applied, we did not identify human studies directly evaluating measured metabolites against validated sleep-, pain- or urgency-specific PROMs; the evidence captured was insufficient to establish signatures for stool-frequency burden or multidimensional symptom clusters. These findings are limited to the scope of this critical narrative review and do not establish that no such studies exist. Importantly, we did not identify any metabolite-related signature that had undergone both analytical and clinical validation for the assessment of patient-reported burden in IBD.

The available human literature uses different biological matrices, analytical platforms and conceptual links to metabolism. Some studies directly measured metabolites in serum, plasma or feces. Others used exploratory multi-omics designs that combined metabolomics with microbiome and proteomic layers. A further group inferred microbial metabolic functions from metagenomic data without direct metabolite quantification. Interventional pathway work is especially informative because it tests whether biochemical pathway modulation translates into symptomatic benefit, and our structured search identified only a small number of such studies.

Table 1 summarizes the design, study population, IBD phenotype and inflammatory activity, PRO or PROM assessment, biological matrix, metabolite-related methodology, principal findings, evidence category, and integrated critical appraisal of the key human studies informing the narrative synthesis. These publications were selected because they evaluated metabolite-related measures and patient-reported symptom burden within the same clinical cohort or intervention, or because they tested a clearly defined metabolic-pathway hypothesis. The table also highlights study-level limitations related to confounding, analytical validity, multiplicity, overfitting, and the absence of independent validation. All eight studies meeting the operational criteria for the principal human evidence synthesis are included in Table 1; Table 1 is therefore complete for that synthesis, although it does not catalogue the broader mechanistic and contextual literature considered elsewhere in this narrative review.

**Figure 1:**
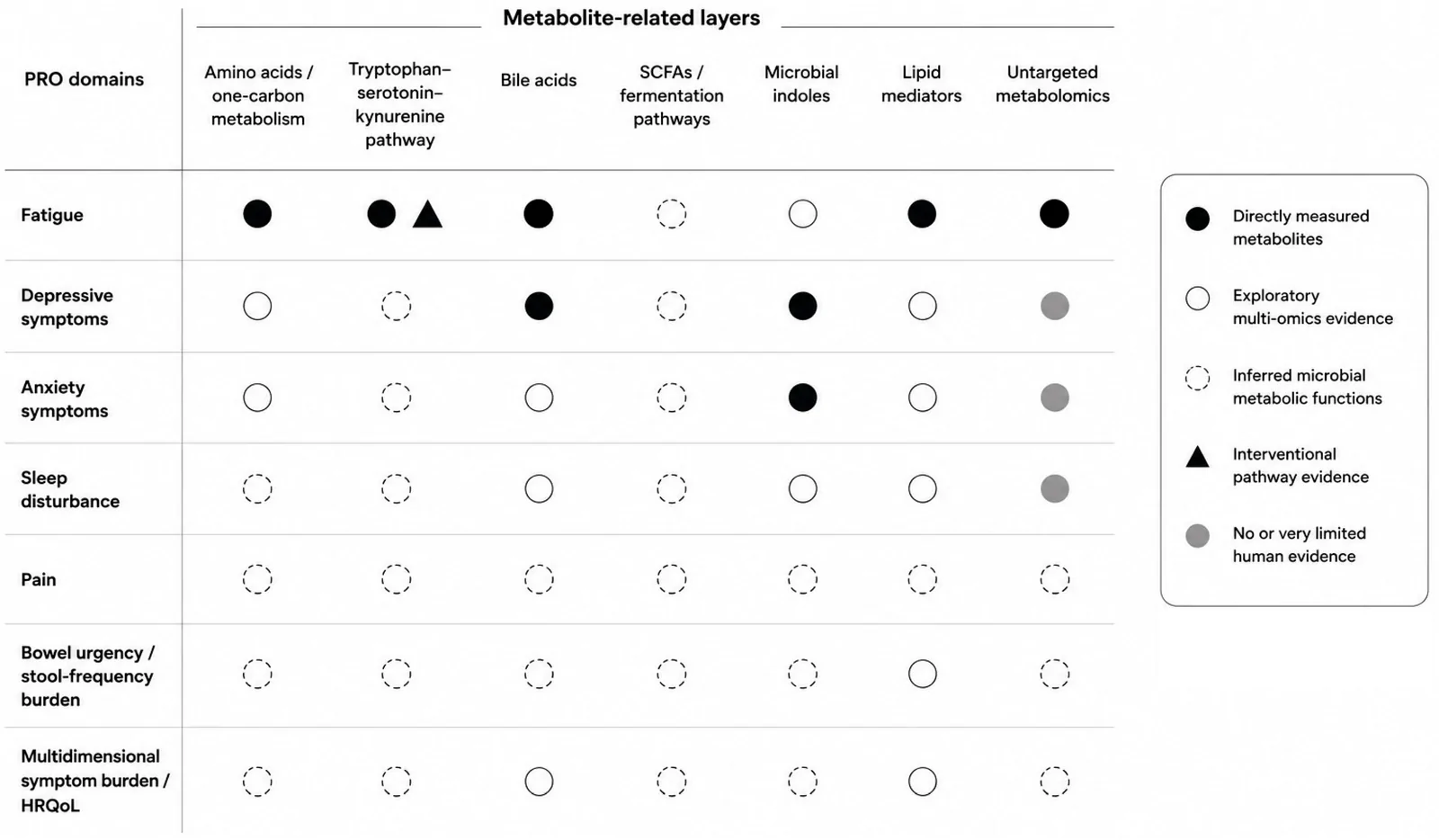
Evidence landscape of metabolite-related measures across patient-reported outcome domains in inflammatory bowel disease. The map distinguishes directly measured metabolites, exploratory multi-omics evidence, inferred microbial metabolic functions, interventional pathway evidence, and mechanistic plausibility. The human studies identified in this review were concentrated primarily in fatigue and questionnaire-defined mood-related symptom burden. For fatigue, evidence based on directly measured metabolites included serum metabolites, plasma lipidomics and fecal untargeted metabolomics, with some studies integrating microbiome profiles. Within the limits of the databases searched, the search strategy and the eligibility criteria applied, we did not identify human studies that directly evaluated measured metabolites against validated sleep-, pain- or urgency-specific PROMs; the evidence captured was insufficient to establish signatures for stool-frequency or multidimensional symptom burden. Within the same scope, we did not identify a metabolite-informed PRO tool that had undergone both analytical and clinical validation. This qualitative evidence map reflects the authors’ narrative assessment: categories describe evidence type, not quality or certainty; “direct” denotes measured metabolites, whereas predicted functions and pathway enrichments indicate potential metabolic capacity or biological context, not metabolite concentrations, pathway activity or flux.

At the patient-reported outcome domain level, the studies identified in this review were unevenly distributed. Figure 1 provides a visual evidence map linking the principal PRO domains with the metabolite-related layers discussed in this review, while distinguishing direct metabolite measurements, exploratory multi-omics evidence, inferred microbial metabolic functions, interventional pathway evidence, and mechanistic plausibility.

### 3.2. Fatigue and Energy-Related Burden

Among the studies identified in this review, fatigue was the symptom domain with the most developed metabolite-related human literature. In quiescent IBD, Borren et al. associated fatigue with a serum metabolite cluster and fecal microbiome alterations [20]. Lower serum methionine, tryptophan, proline and sarcosine, together with depletion of butyrate-associated taxa, support a candidate host-microbe metabolic context for fatigue beyond overt mucosal activity. These findings remain exploratory.

Horta et al. examined plasma lipidomic profiles in 47 patients with quiescent IBD (27 CD and 20 UC; 23 with fatigue and 24 without fatigue) using the FACIT-FS and UPLC-TOFMS [21]. Fatigued participants had lower levels of phosphatidylcholines, plasmanyls, sphingomyelins, lysophosphatidylcholines, phosphatidylethanolamines, phosphatidylinositols, phosphatidylserines and eicosanoids. Pathway analyses implicated arachidonic-acid and glycerophospholipid metabolism and the sphingolipid pathway. This study linked directly measured plasma lipids with a fatigue PROM, but the association remains exploratory.

Liu et al. compared four groups of 30 participants—UC with fatigue, UC without fatigue, healthy participants with fatigue and healthy participants without fatigue—using Fatigue Scale-14 (FS-14; fatigue defined as a score ≥ 7), untargeted fecal LC–MS/MS and 16S rRNA profiling [22]. Compared with UC without fatigue, UC with fatigue showed 262 differential fecal metabolites; linoleoyl ethanolamide differed between groups, arachidonoyl ethanolamide and glycocholic acid were increased, and thromboxane was detected only in the UC-with-fatigue group. Eicosanoid, tryptophan and tyrosine pathways were implicated. This study linked directly measured fecal metabolites with fatigue; interpretation remains limited by its cross-sectional design and numerical imbalance in active or severe disease.

In active IBD, Thomann et al. linked fatigue and HADS-D depressive symptom severity to fecal metagenomic features and predicted functional modules related to amino-acid metabolism, methionine biosynthesis, central carbohydrate metabolism, pentose-phosphate pathways and SCFA-related taxa [23]. This work is important because it moves beyond taxonomy, but the metabolic interpretation remains inferred microbial metabolic potential rather than directly measured metabolite concentration or metabolic flux.

Interventional pathway evidence is especially valuable because it tests whether biochemical pathway modulation changes patient-reported outcomes. In quiescent IBD with fatigue, 5-hydroxytryptophan (5-HTP) increased serum 5-hydroxytryptophan and serotonin concentrations but did not improve fatigue or mood/stress-related PROs compared with placebo [27]. This result should be interpreted as a negative finding for this specific peripheral 5-HTP supplementation strategy and study population, not as evidence against the broader involvement of tryptophan-kynurenine metabolism, central serotonergic signaling, phenotype-specific pathway effects, or alternative approaches to modulating this pathway.

### 3.3. Mood-Related Burden: Depression and Anxiety

Among the studies identified in this review, mood-related burden was another relatively developed domain, but terminology requires caution. Studies using PHQ-9, GAD-7 or other scales assess depressive symptoms, anxiety symptoms or questionnaire-defined psychological burden; they do not necessarily establish clinical diagnoses of major depression or anxiety disorder.

Yuan et al. integrated gut microbiota, serum metabolomics and proteomics in active ulcerative colitis and identified a multi-omics network associated with PHQ-9/GAD-7-defined depressive and anxiety symptoms [24]. Increased glycochenodeoxycholate and altered levels of metabolites such as 2′-deoxy-D-ribose and L-pipecolic acid suggest that mood-related symptom burden may co-occur with microbial, metabolic, immune and inflammatory layers. However, active disease and systemic inflammation may be common causes, mediators or downstream consequences within this network; animal experiments from the same report should be treated as mechanistic context rather than direct human validation.

Feng et al. associated gut microbiota dysbiosis and fecal and serum bile-acid alterations with questionnaire-defined psychological symptom burden in Crohn’s disease [25]. Wang et al. subsequently linked HADS-defined anxiety and depressive symptoms in IBD with fecal microbiota, Tax4Fun2-predicted functional pathways and untargeted fecal LC–MS metabolites, including 3-methyl-2-oxobutanoic acid, alpha-ketoglutaric acid, pipecolic acid, glycocholic acid, taurochenodeoxycholic acid and indole-3-acetic acid [26]. Together, these studies suggest bile-acid, amino-acid, butanoate and tryptophan-related pathways for further study, but remain exploratory.

Taxonomy-only studies provide useful mechanistic or contextual evidence when interpreted cautiously. Humbel et al. associated intestinal microbiota alterations with impaired psychological function in patients with IBD in remission [28]. Scaldaferri et al. reported gut microbiota signatures associated with psychopathological profiles in ulcerative colitis [29]. Such studies provide mechanistic or contextual evidence rather than evidence based on direct metabolite measurement, because metabolites or metabolite-associated functional pathways were not assessed in the same clinical context.

### 3.4. Sleep Disturbance

Current human evidence. Within the limits of the databases searched, the search strategy and the eligibility criteria applied, we did not identify human studies directly evaluating associations between measured metabolites and validated sleep PROMs in IBD. PROM-focused studies show that insomnia is common and associated with mental health conditions and IBD activity [30], but these studies generally do not include metabolomics or microbial metabolic-function assessment in the same participants.

Interpretative context. Sleep disturbance may plausibly involve tryptophan-serotonin-kynurenine metabolism, inflammatory signaling, corticosteroid exposure, circadian disruption, autonomic activation and altered activity patterns. Wearable physiology should be presented only as a potential future integration layer for longitudinal symptom and activity monitoring, not as metabolite-PRO evidence [31].

Evidence gap. The mechanistic and PROM-only studies identified in this review provide insufficient evidence to validate these pathways against sleep-specific PROs or objective sleep measures in well-phenotyped IBD cohorts. Among studies captured by this search, we did not identify any metabolite-related sleep signature that had undergone clinical validation for the assessment of sleep burden.

Research priority. Future studies should combine validated sleep PROMs, actigraphy or wearable-derived sleep metrics, inflammatory markers, steroid and caffeine exposure, medication metadata, diet, repeated sampling and targeted tryptophan–serotonin–kynurenine metabolite panels.

### 3.5. Pain

Current human evidence. Within the limits of the databases searched, the search strategy and the eligibility criteria applied, we did not identify human studies directly evaluating associations between measured metabolites and validated pain-specific PROMs in IBD. Pain is often embedded within global symptom indices or health-related quality-of-life instruments, limiting interpretation of metabolite-pain associations.

Interpretative context. Pain is biologically plausible in relation to lipid mediators, bile acids, barrier dysfunction, immune activation, mast-cell signaling, visceral hypersensitivity and neuroimmune pathways. Lipidomics is particularly relevant because eicosanoids, oxylipins, sphingolipids and membrane lipids participate in inflammatory signaling and nociception [32].

Evidence gap. The studies identified in this review provide insufficient evidence to determine whether specific metabolite-related signatures distinguish inflammatory pain, irritable bowel syndrome (IBS) overlap, visceral hypersensitivity, bile-acid diarrhea, fibrostenotic complications, pelvic-floor dysfunction or medication-related pain. Among studies captured by this search, we did not identify any pain-specific metabolite signature that had undergone clinical validation against a pain-specific PROM.

Research priority. Pain-focused cohorts should stratify by inflammatory activity, disease location, stricturing/penetrating complications, IBS overlap, stool form, bile-acid malabsorption risk, non-steroidal anti-inflammatory drug (NSAID)/opioid exposure and psychological burden, while using pain-specific PROMs and targeted lipid mediator or bile-acid panels.

### 3.6. Bowel Urgency, Stool Frequency, and Bowel Confidence

Current human evidence. Within the limits of the databases searched, the search strategy and the eligibility criteria applied, we did not identify studies directly pairing measured metabolites with validated urgency-specific or bowel-confidence PROMs. Serum bile-acid metabolites have been studied as predictors of mesalazine response [33], and altered fecal bile-acid composition has been reported in active UC [34], but neither study addressed urgency-specific burden.

Interpretative context. The clinical rationale for bile-acid involvement is strongest in ileal CD, ileal resection and bile-acid malabsorption; the underlying pathways are summarized in Section 4.2. Fecal concentrations are influenced by transit, ileal absorption and stool water content and should not be interpreted as direct measures of pathway activity.

Research priority. Future studies should pair targeted serum or fecal bile-acid panels with validated urgency and stool-frequency instruments in phenotype-stratified UC and ileal CD cohorts.

### 3.7. Multidimensional Symptom Clusters and Health-Related Quality of Life

Current human evidence. Symptom-cluster studies describe co-occurring fatigue, pain, sleep disturbance, bowel symptoms and psychological distress [35,36,37], while IBSEN III documents fatigue across disease stages [38,39]. Within the search scope and eligibility criteria, the identified studies characterized patient-reported burden but did not align multidimensional phenotypes with directly measured metabolite profiles.

Interpretative context and research priority. Multidimensional burden may be more biologically realistic than one-symptom/one-metabolite models. Future studies should combine validated PROM clusters with repeated metabolomics and latent-profile or network analyses; domain-specific design priorities are summarized in Table 2.

## 4. Metabolite Classes and Their Potential Relevance to Patient-Reported Burden

### 4.1. Short-Chain Fatty Acids

SCFAs are generated mainly through bacterial fermentation of non-digestible carbohydrates. Systematic review evidence summarizes therapeutic and immunologic interest in SCFAs in IBD [40]. SCFAs can regulate epithelial and immune pathways relevant to IBD biology [41]. Quantitative alterations in SCFAs have been reported across IBD studies [42]. Stool SCFA levels have also been measured directly in patients with IBD and healthy controls [43]. Broader host-microbiota literature supports the importance of SCFA-related interactions in chronic disease biology [44]. These mechanisms make SCFAs plausible contributors to barrier-related symptoms, bowel burden and fatigue-related biology.

Stool SCFA concentrations integrate production, absorption, microbial and colonocyte utilization, transit and stool water content; they therefore cannot be interpreted as production rates. Absolute quantification and matrix-specific metadata are essential.

### 4.2. Bile Acids

Bile acids are host-microbial signaling molecules transformed through hepatic synthesis, intestinal conjugation and microbial deconjugation. IBD studies have examined serum metabolites as predictors of mesalazine response [33], fecal composition in active UC [34], FXR/TGR5 signaling [45,46], nutritional and microbial modulation [47] and anti-integrin response in experimental colitis [48].

Their most plausible PRO relevance concerns urgency and diarrhea in ileal CD or after ileal resection, and questionnaire-defined mood-related burden in CD [25]. Interpretation should preserve species-level and matrix-specific distinctions; study-design priorities are summarized in Table 2.

### 4.3. Tryptophan, Kynurenine, Serotonin, and Microbial Indoles

Tryptophan metabolism connects inflammation, microbial metabolism and neuroactive signaling. Tryptophan can enter the kynurenine pathway, serotonin-related metabolism or microbial indole production. Transcriptomic and metabolomic synthesis has implicated intestinal tryptophan metabolism in IBD [49]. Mendelian randomization work has also examined the tryptophan-kynurenine pathway in relation to IBD [50]. Immune activation can induce indoleamine 2,3-dioxygenase and shift metabolism toward kynurenine-pathway products, providing a plausible link between inflammation, fatigue, low mood and immune-metabolic regulation.

The 5-HTP trial in quiescent IBD is an important example of how metabolic-pathway plausibility requires clinical testing [27]. Oral 5-HTP increased serum 5-HTP and serotonin, indicating a peripheral biochemical response, but this did not translate into improvement in fatigue or mood-related secondary outcomes in that population. This finding should not be interpreted as evidence against tryptophan-kynurenine biology, central serotonergic signaling, microbial indole pathways, phenotype-specific effects or other approaches to modulating this axis. Aryl hydrocarbon receptor activation has been discussed as a microbiota-linked pathway in IBD [51]. Gut microbiome-related anti-inflammatory effects of aryl hydrocarbon receptor activation also provide mechanistic context for IBD biology [52]. We did not identify human studies directly evaluating associations between measured indole/AhR-related metabolites and validated IBD symptom PROMs.

### 4.4. Lipid Mediators and Lipidomic Signatures

Lipid mediators, including eicosanoids, oxylipins, sphingolipids and membrane lipids, are relevant to inflammatory signaling, epithelial stress, nociception and systemic symptom burden. Lipidomics reviews support the importance of lipid pathways in IBD pathogenesis [32].

Horta et al. linked directly measured plasma lipidomic patterns with fatigue in quiescent IBD, illustrating a clinically relevant discovery signal that still requires independent validation [21].

Lipidomic interpretation requires careful distinction between targeted and untargeted approaches, annotation confidence, reference standards, internal standards, drift correction and batch correction. Without these elements, lipid features may be useful for discovery but remain difficult to translate into clinically interpretable patient-reported burden signatures.

### 4.5. Volatile Organic Compounds and Untargeted Metabolomic Signatures

VOCs and broad untargeted metabolomic profiles may offer composite signatures of host-microbial metabolism, diet, transit and inflammation. Fecal volatile organic metabolites have been investigated as potential diagnostic biomarkers in IBD [53]. Liu et al. directly paired untargeted fecal LC–MS/MS with FS-14-defined fatigue in UC [22], whereas broader untargeted fecal metabolomics has also been used to discover biomarker and treatment-target candidates in IBD [54]. We did not identify human IBD studies directly pairing VOC profiles with validated PROMs. VOCs should therefore be framed primarily as a potential future research direction unless studies directly pair VOC profiles with validated patient-reported outcomes.

Untargeted metabolomics should therefore report analytical platform, metabolite identification level, quality-control samples, internal standards, randomization of analysis order, missing-data handling, normalization, multiple-testing control and feature annotation confidence. Candidate features require confirmation with reference standards and independent validation before they can be interpreted as PRO-related signatures.

## 5. Methodological and Translational Challenges

Table 1 presents the study-level appraisal; the following sections focus on the cross-cutting issues most relevant to interpretation and translation.

### 5.1. Disease Activity and Phenotype Confounding

Inflammation can be a confounder, mediator, effect modifier or common cause in metabolite-PRO relationships. It may simultaneously alter the metabolome and produce symptoms, act through metabolic pathways, be modified by metabolites, influence diet, treatment, sleep and activity, or interact bidirectionally with psychological symptom burden. Simple statistical adjustment for C-reactive protein (CRP) or fecal calprotectin may therefore be inappropriate when inflammation lies on the causal pathway. Model covariates should be chosen according to an explicit causal hypothesis rather than added mechanically.

IBD phenotype is equally important. Ileal Crohn’s disease, colonic Crohn’s disease, ulcerative colitis, pouch status, ileal resection and stricturing or penetrating behavior may produce different metabolic profiles. Mixed cohorts without stratification can obscure location-specific bile-acid and fermentation effects, and reported Crohn’s disease/ulcerative colitis structure should be preserved when interpreting studies.

### 5.2. Heterogeneity of PROs and PROMs

PROM heterogeneity limits interpretability. Future studies should name and justify instruments, for example FACIT-F or PROMIS-Fatigue for fatigue, HADS or PHQ-9/GAD-7 for depressive and anxiety symptoms, validated sleep scales or actigraphy-supported sleep measures, pain numeric rating scales or disease-specific pain items, and urgency/stool-frequency instruments. Psychological symptom burden should not be conflated with formal psychiatric diagnosis unless diagnostic assessment was performed. PROM choice should also consider responsiveness, defined as the ability of an instrument to detect clinically meaningful change over time, and floor or ceiling effects, which occur when scores cluster at the lower or upper end of the scale and limit detection of worsening or improvement.

Pooling fatigue, pain, depressive symptoms, sleep disturbance and quality of life into a generic burden score may be clinically convenient but biologically imprecise. Symptom-domain specificity is essential for metabolite interpretation.

### 5.3. Biological Matrices and Pre-Analytical Variability

Stool, serum, plasma, urine and mucosal biopsies address different biological questions. Stool is suited to luminal fermentation, bile-acid transformation and microbial metabolic output; serum or plasma may better reflect systemic fatigue and host-integrated metabolism; biopsies capture tissue-proximal mucosal metabolism but are invasive and location-dependent.

Pre-analytical variability is central in metabolomics. Studies should report fasting status, time of day, time to freezing, storage temperature, freeze-thaw cycles, stool form, stool water content, extraction methods and bowel preparation or antibiotic exposure. For fecal metabolites, transit time and water content can strongly influence concentration, and fecal concentration should not be interpreted simplistically as production rate. This principle applies not only to SCFAs but also to bile acids, amino-acid derivatives, indoles and other fecal metabolites.

### 5.4. Analytical Platforms and Metabolite Identification

Targeted and untargeted metabolomics answer different questions. Targeted LC–MS/MS, GC–MS and NMR approaches can quantify predefined metabolites, sometimes with absolute concentrations when reference standards and internal standards are used. Untargeted approaches provide broader discovery potential but generate many unknown or partially annotated features.

For metabolomics-focused research, analytical transparency is essential and should be evaluated at the study level. Studies should report the platform used, chromatographic conditions where relevant, relative versus absolute quantification, metabolite identification level, reference standards, pooled quality-control samples, internal standards, randomization of analysis order, instrument drift, batch correction, normalization procedures and missing-data handling. Biological conclusions should distinguish an analytical feature, a tentatively annotated compound, a putatively identified metabolite and a metabolite confirmed with a reference standard.

### 5.5. Statistical Validity, Multiple Testing, and Overfitting

Metabolomics and microbiome studies often combine high-dimensional data with modest sample sizes. This creates risks of false discovery, overfitting, data leakage and unstable feature selection. Here, high-dimensional data contain many measured features, often approaching or exceeding the number of participants. Overfitting occurs when a model captures sample-specific noise rather than reproducible signal; data leakage occurs when information from validation or test data influences model development; and unstable feature selection means that small changes in the sample or analysis lead to different features being selected. Future studies should pre-specify primary symptom domains, apply false-discovery-rate correction, separate discovery and validation sets where feasible, report model calibration as well as discrimination, and validate candidate signatures externally.

### 5.6. Causality and Directionality

**Figure 2:**
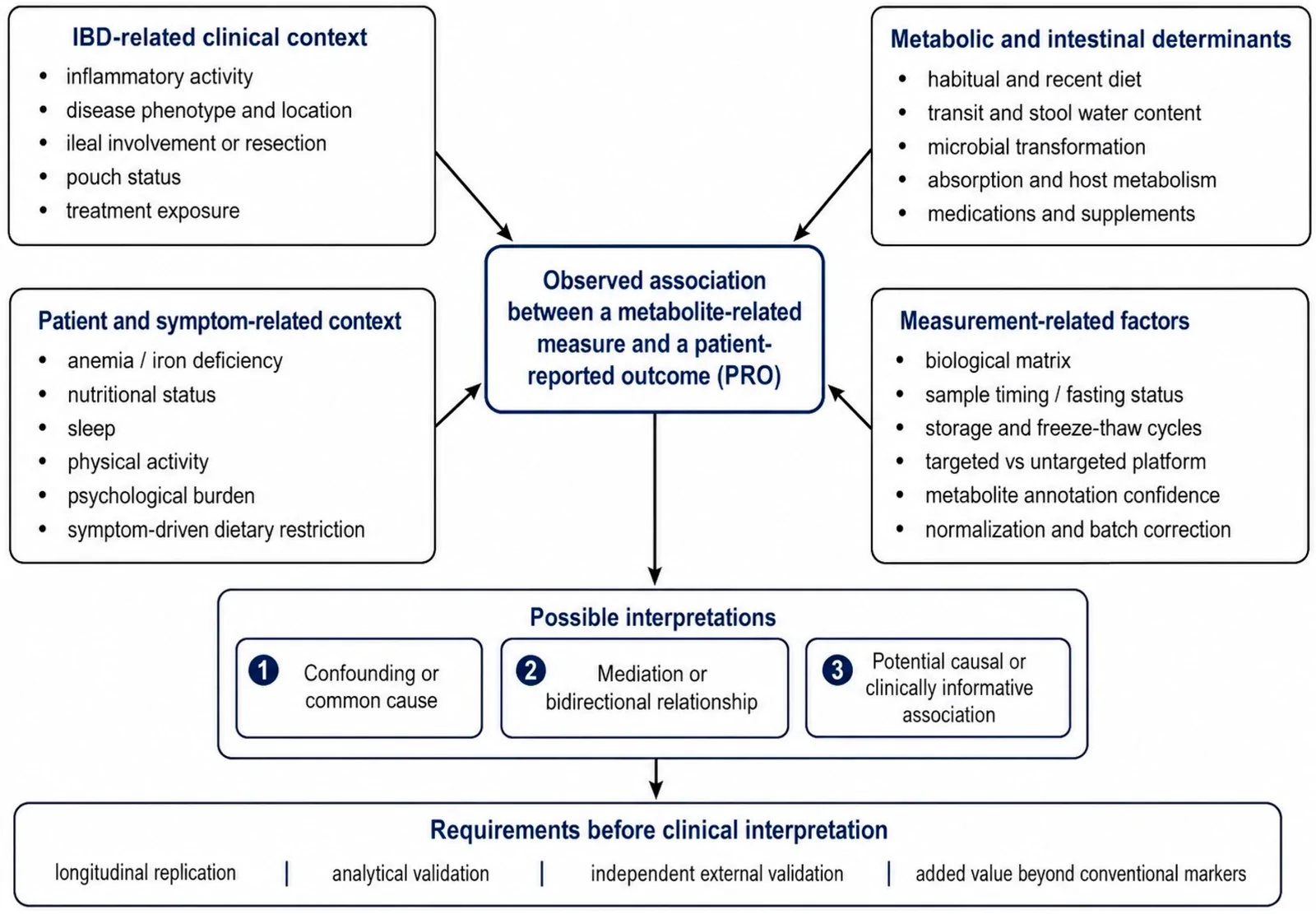
Conceptual Framework for the Interpretation of Metabolite–Patient-Reported Outcome Associations in Inflammatory Bowel Disease. The framework is author-proposed and represents a research agenda for interpreting candidate metabolite–PRO associations rather than a validated clinical tool.

Metabolite-PRO associations may reflect several causal structures: a metabolite may be associated with a symptom, a symptom may change diet and behavior and thereby alter the metabolome, inflammation may be associated with both, medication may be associated with both, or transit and disease location may confound both. Diet may act as a determinant of metabolite concentration, a confounder, a mediator of symptom effects on the metabolome, a therapeutic exposure or an effect modifier of microbiome-PRO relationships. Future studies should collect current and habitual diet, supplementation, fiber intake, enteral nutrition, elimination diets and timing between dietary assessment, biospecimen collection and PROM completion. Figure 2 summarizes the principal clinical, patient-related, intestinal, metabolic, pre-analytical, and analytical factors that may influence an observed association between a metabolite-related measure and a PRO. These factors may act as confounders, mediators, effect modifiers, common causes, or consequences of patient-reported symptoms and should be considered before causal or clinical interpretation.

### 5.7. Reproducibility, Validation, and Clinical Context of Use

Translational requirements depend on the intended context of use. Mechanistic phenotyping requires adequate analytical performance and reproducibility. Cross-sectional classification additionally requires prespecified model development and independent external validation in representative cohorts, but cross-sectional validation does not establish longitudinal predictive performance. Prognostic and treatment-response prediction require prospective longitudinal validation with temporally ordered assessment of predictors and outcomes. Longitudinal monitoring additionally requires evidence of within-person responsiveness to changes in symptom burden. Clinical decision-support applications further require demonstration of incremental value beyond PROMs and conventional clinical markers and, ultimately, evidence that their use improves interpretation or clinical decision-making. Figure 2 summarizes the principal sources of interpretative uncertainty relevant to these applications.

## 6. Research Priorities

Table 2 consolidates domain-specific candidate metabolite layers, current human support, interpretative cautions and priority study designs. Near-term work should replicate the candidate fatigue- and mood-related associations for which preliminary human evidence exists, whereas sleep disturbance, pain, bowel urgency/stool frequency and multidimensional symptom burden require phenotype-specific discovery studies using validated domain-specific PROMs. Across domains, studies should prioritize prospective, preferably multicenter designs, synchronize PROM completion with biospecimen collection and incorporate repeated longitudinal assessment when monitoring, prognosis or treatment-response prediction is intended. Protocols should standardize the biological matrix, sampling time and pre-analytical handling and document key clinical and behavioral determinants, including inflammatory activity, disease phenotype, diet, medication exposure, sleep and physical activity. Analyses should pre-specify hypotheses, distinguish confounders, mediators and effect modifiers, control multiplicity, prevent data leakage and report null findings. Candidate signals should undergo analytical validation and independent external validation in representative cohorts before evaluation of incremental clinical value; monitoring applications additionally require demonstration of within-person responsiveness.

## 7. Strengths and Limitations of the Narrative Review

A major strength of this review is the structured separation of evidence layers and explicit attention to PROM definition, analytical method and translational maturity, consistent with SANRA principles.

Several limitations should be considered. This critical narrative review used a structured search to support purposive synthesis rather than exhaustive study enumeration, so source selection may reflect author judgment. No formal risk-of-bias or certainty-of-evidence framework was applied. Substantial heterogeneity in IBD phenotype, inflammatory activity, biological matrix, analytical platform and PROM definition limited cross-study comparison, and the predominance of cross-sectional designs constrained causal interpretation.

## 8. Conclusions

Human evidence was concentrated in fatigue and questionnaire-defined mood-related symptom burden. Fatigue studies included targeted serum metabolomics, plasma lipidomics and fecal untargeted metabolomics based on directly measured metabolites, predicted microbial functions and a 5-HTP pathway trial without symptomatic benefit. Mood-related studies included serum and fecal metabolomics, fecal bile-acid profiling and multi-omics networks. Within the limits of the databases searched, the search strategy and the eligibility criteria applied, we did not identify studies pairing directly measured metabolites with PROMs for sleep, pain or urgency, and the evidence captured for multidimensional clusters was insufficient.

These findings support candidate associations and exploratory signatures, including directly measured signals, but not clinically actionable biomarkers. Translation will require context-specific analytical and clinical validation and demonstration of added value beyond PROMs and conventional clinical markers.

## Figures and Tables

**Table 2:** Integrated Metabolite Classes, PRO Domains and Research Priorities for Future Metabolite-PRO Studies in IBD.

Focus Area	Candidate Metabolite Layers	Current Human Support	Key Interpretative Cautions	Priority Study Design
Fatigue and energy-related burden	Serum amino-acid-related metabolites, plasma lipid classes and lipid mediators, fecal untargeted metabolomic features, SCFA-related microbiome features, tryptophan-serotonin pathway measures.	Targeted serum metabolomics, plasma lipidomics and fecal untargeted metabolomics based on directly measured metabolites; inferred microbial functions; and a 5-HTP pathway trial without symptomatic benefit.	Fatigue is multifactorial and overlaps with anemia, sleep disturbance, mood and inflammatory activity.	Repeated-measures active/quiescent cohorts with validated fatigue PROMs, synchronized biospecimens and independent validation.
Questionnaire-defined mood-related symptom burden	Serum/fecal metabolomics, bile acids, amino-acid pathways, tryptophan/indole metabolism, microbiome-derived functional potential.	Serum and fecal metabolomics and fecal/serum bile-acid profiling based on directly measured metabolites; exploratory multi-omics and inferred microbial functional context linked to questionnaire-defined symptoms.	Screening-scale positivity is not a psychiatric diagnosis; active inflammation complicates interpretation.	Prospective cohorts separating screening-scale positivity from diagnosis and pairing symptom assessment with serum/fecal metabolomics.
Sleep disturbance	Tryptophan-kynurenine-serotonin metabolism, inflammatory metabolites, circadian and steroid-related metabolic context.	No study pairing directly measured metabolites with a validated sleep PROM was identified.	Wearables are future integration tools, not metabolite-PRO evidence.	Validated sleep PROMs plus actigraphy and repeated tryptophan-pathway panels.
Pain	Lipid mediators, oxylipins, sphingolipids, bile acids, barrier-related metabolites.	No study pairing directly measured metabolites with a pain-specific PROM was identified.	Pain phenotypes vary across inflammation, IBS overlap, structural complications and visceral hypersensitivity.	Phenotype-stratified cohorts with pain-specific PROMs and targeted lipid mediator/bile-acid panels.
Bowel urgency, stool frequency and bowel confidence	Primary/secondary bile acids, conjugated/unconjugated bile-acid species, SCFAs and fermentation-related metabolites.	Mechanistic and disease-activity evidence; no study pairing directly measured metabolites with a validated urgency PROM was identified.	Fecal concentration is not pathway production; ileal disease/resection, transit, stool water and sequestrants are major determinants.	UC and ileal-CD cohorts pairing targeted bile-acid panels with validated urgency/stool-frequency instruments.
Multidimensional symptom burden and HRQoL	Composite targeted/untargeted metabolomics, microbiome function, diet-related and host-response signatures.	No directly measured metabolite signature was established for validated multidimensional symptom clusters.	HRQoL is broad and not equivalent to symptom-specific PRO; overfitting risk is high in multi-domain models.	Repeated-sampling latent-profile or network analyses with discovery-validation splits.

Scope note: Statements that evidence was not identified or established are limited to the databases, search strategy and eligibility criteria used in this critical narrative review. Evidence-layer note: Predicted microbial functions, metabolite-associated taxa and pathway-enrichment analyses indicate potential metabolic capacity or biological context, not metabolite concentrations, pathway activity or metabolic flux. Abbreviations: 5-HTP, 5-hydroxytryptophan; CD, Crohn’s disease; HRQoL, health-related quality of life; IBD, inflammatory bowel disease; PRO, patient-reported outcome; PROM, patient-reported outcome measure; SCFA, short-chain fatty acid; UC, ulcerative colitis.

## Data Availability

No new data were created or analyzed in this study. Data sharing is not applicable to this article.

## Supplementary Materials

The following supporting information can be downloaded at: https://www.mdpi.com/article/10.3390/metabo16090696/s1, Table S1: Comparison of the Scope and Methodological Focus of Previous Reviews and the Present Critical Narrative Review [8,9,10,14,15,16,17,18]; Table S2: Database-Specific Search Strategies Used for Literature Identification.
