# Supplementary material for: Metabolite-Related Evidence Linking Metabolic Measures to Patient-Reported Outcomes in Inflammatory Bowel Disease: A Critical Narrative Review
Source: Metabolites. 2026 Sep 20;16(9):696. doi: 10.3390/metabo16090696 (PMC13608916; doi:10.3390/metabo16090696)
Supplement: Supplementary file 1 [file metabolites-16-00696-s001.zip › metabolites-4559020-supplementary.pdf]

Table S1. Comparison of the Scope and Methodological Focus of Previous Reviews and the Present Critical Narrative Review

Review	*Primary focus*	*PROs or patient-reported symptom domains as a central focus*	*Direct metabolite measurements distinguished from inferred microbial functions*	*Different evidence layers explicitly separated*	*Study-level appraisal of metabolite–PRO evidence*	*Main distinction from the present review*
Banfi et al., 2021 [10]	Microbial metabolites and the microbiota–gut–brain axis in IBD, including gastrointestinal and neuropsychiatric mechanisms and microbiome-targeted therapies	Partial; gastrointestinal and neuropsychiatric symptoms were discussed, but validated PROs and PROMs were not the principal organizing framework	Partial; major microbial metabolite classes were discussed, but measured metabolites were not consistently separated from microbial composition and mechanistic evidence	No formal separation into direct measurements, inferred functions, multi-omics, and interventional evidence	No dedicated study-level appraisal focused on metabolite–PRO associations	Mechanistic gut–brain-axis review rather than a PRO-centered evaluation of the clinical evidence
Gallagher et al., 2021 [14]	Systematic review of metabolomic alterations in IBD, including diagnostic, disease-activity, and pathophysiological applications	No; PROs and symptom-specific PROMs were not the primary clinical phenotypes	Not applicable as a central objective; the review focused predominantly on studies using direct metabolomic measurements	No PRO-specific stratification of evidence layers	Methodological limitations of metabolomics studies were considered, but not specifically in relation to PRO assessment	Comprehensive metabolomics synthesis without a specific focus on patient-reported burden
Aldars-García et al., 2021 [15]	Broad overview of metabolomics in IBD, including pathogenesis, diagnosis, prognosis, disease activity, and treatment response	No; patient-reported symptom domains were not the principal focus	Partial; metabolomic findings were described, but their relationship to inferred microbial metabolic functions was not the main methodological distinction	No systematic separation of evidence according to its proximity to direct metabolite–PRO measurement	No dedicated appraisal of metabolite–PROM validity or clinical context of use	Broad metabolomics review rather than a symptom-domain-specific and PRO-centered synthesis
Truyens et al., 2024 [8]	Pathogenesis and management of IBD-related fatigue, including inflammatory, psychological, nutritional, and gut–brain-axis mechanisms	Yes, but limited primarily to fatigue	Partial; metabolites, microbial features, and mechanistic pathways were discussed, but direct and inferred metabolic evidence were not consistently evaluated as separate categories	Partial; observational and interventional findings were discussed, but not within a formal metabolite-evidence framework	No comprehensive appraisal of metabolite-related evidence across multiple PRO domains	Fatigue-specific review rather than a broader synthesis of metabolite-related evidence across different PROs
Boldyreva et al., 2024 [9]	Broad and emerging mechanisms underlying bidirectional gut–brain communication in IBD	Partial; psychological and neurological burden was discussed mainly as part of the mechanistic context	No systematic distinction between directly measured metabolites and inferred or mechanistic metabolic pathways	No formal evidence-layer classification	No study-level appraisal centered on metabolite–PRO associations	Broad mechanistic gut–brain-axis perspective without a validated PROM-centered framework
Shen et al., 2025 [16]	Gut microbiota and microbiota-derived metabolites as mechanistic and therapeutic targets in IBD	No; symptoms and clinical outcomes were discussed mainly in relation to inflammation and therapeutic response	Partial; metabolite classes were described, but direct measurement, predicted function, and mechanistic evidence were not consistently separated	No formal separation of direct, inferred, multi-omics, and interventional metabolite–PRO evidence	No dedicated appraisal of PRO measurement, confounding, or clinical validation	Therapeutic and mechanistic review rather than an assessment of metabolite-related correlates of patient-reported burden
Zarei et al., 2025 [17]	Gut-microbial metabolites and anxiety/depression in IBD.	Partial; no broader PRO/PROM framework.	No systematic direct-versus-inferred study-level distinction.	No formal separation of evidence layers.	No structured appraisal of PROM validity, diagnosis, confounding, multiplicity or validation.	Present review spans fatigue, mood, sleep, pain and bowel symptoms and distinguishes screening burden from psychiatric diagnosis.
Sharkiya et al., 2026 [18]	Systematic review of metabolomics for IBD diagnosis, disease differentiation, activity assessment, treatment response and monitoring.	No; PROs/PROMs were not the organizing framework.	Predominantly direct metabolomics; no PRO-specific direct-versus-inferred classification.	No PRO-specific separation of evidence layers.	Systematic appraisal of metabolomics studies, not metabolite–PRO measurement validity.	Latest broad metabolomics review; the present review is PRO-centered and symptom-domain-specific.
Present review	Metabolite-related measures and metabolic signals associated with IBD PROs.	Yes; fatigue, mood, sleep, pain and bowel-symptom burden.	Yes; direct measurements and inferred functions are separated.	Yes; direct, multi-omics, inferred, interventional and mechanistic evidence are separated.	Yes; PROM/method rigor, confounding, multiplicity, validation.	PRO-centered appraisal of evidence maturity and clinical translatability.

Abbreviations: PRO, patient-reported outcome; PROM, patient-reported outcome measure; IBD, inflammatory bowel disease.

Table S2. Database-Specific Search Strategies Used for Literature Identification

Searches were conducted up to 11 June 2026. No publication-date, language, age, species, or study-design filters were applied at the database-search stage. The search strategies supported a critical narrative review and purposive narrative synthesis rather than a formal systematic-review screening process.

Database / Platform	*Date and Limits*	*Database-Specific Search Strategy*
PubMed/MEDLINE	11 June 2026; no date, language, age, species, or study-design filters at the database-search stage.	(("Inflammatory Bowel Diseases"[Mesh] OR "inflammatory bowel disease"[tiab] OR IBD[tiab] OR "Crohn Disease"[Mesh] OR Crohn*[tiab] OR "Ulcerative Colitis"[Mesh] OR "ulcerative colitis"[tiab] OR "IBD-unclassified"[tiab] OR "IBD unclassified"[tiab])<br>AND<br>("Metabolomics"[Mesh] OR metabolomic*[tiab] OR metabolite*[tiab] OR "metabolic signature"[tiab] OR "metabolic signatures"[tiab] OR "microbiota-derived metabolite"[tiab] OR "microbiota-derived metabolites"[tiab] OR "short-chain fatty acid"[tiab] OR "short-chain fatty acids"[tiab] OR SCFA[tiab] OR butyrate[tiab] OR propionate[tiab] OR acetate[tiab] OR "bile acid"[tiab] OR "bile acids"[tiab] OR tryptophan[tiab] OR kynurenine[tiab] OR serotonin[tiab] OR indole*[tiab] OR AhR[tiab] OR "aryl hydrocarbon receptor"[tiab] OR lipidomic*[tiab] OR oxylipin*[tiab] OR sphingolipid*[tiab] OR "volatile organic compound"[tiab] OR "volatile organic compounds"[tiab] OR VOC[tiab] OR "microbial function"[tiab] OR "microbial functions"[tiab] OR metagenom*[tiab])<br>AND<br>("Patient Reported Outcome Measures"[Mesh] OR "patient-reported outcome"[tiab] OR "patient-reported outcomes"[tiab] OR PRO[tiab] OR PROM[tiab] OR "quality of life"[tiab] OR HRQoL[tiab] OR fatigue[tiab] OR tiredness[tiab] OR depression[tiab] OR depressive[tiab] OR anxiety[tiab] OR mood[tiab] OR sleep[tiab] OR insomnia[tiab] OR pain[tiab] OR urgency[tiab] OR "stool frequency"[tiab] OR "symptom burden"[tiab] OR "bowel confidence"[tiab]))
Embase.com	11 June 2026; no date, language, age, species, or study-design filters at the database-search stage.	(('inflammatory bowel disease'/exp OR 'crohn disease'/exp OR 'ulcerative colitis'/exp OR 'inflammatory bowel disease':ti,ab,kw OR ibd:ti,ab,kw OR crohn*:ti,ab,kw OR 'ulcerative colitis':ti,ab,kw OR 'ibd unclassified':ti,ab,kw)<br>AND<br>('metabolomics'/exp OR 'metabolite'/exp OR metabolomic*:ti,ab,kw OR metabolite*:ti,ab,kw OR 'metabolic signature':ti,ab,kw OR 'metabolic signatures':ti,ab,kw OR 'microbiota derived metabolite':ti,ab,kw OR 'microbiota derived metabolites':ti,ab,kw OR 'short chain fatty acid':ti,ab,kw OR 'short chain fatty acids':ti,ab,kw OR scfa:ti,ab,kw OR butyrate:ti,ab,kw OR propionate:ti,ab,kw OR acetate:ti,ab,kw OR 'bile acid':ti,ab,kw OR 'bile acids':ti,ab,kw OR tryptophan:ti,ab,kw OR kynurenine:ti,ab,kw OR serotonin:ti,ab,kw OR indole*:ti,ab,kw OR ahr:ti,ab,kw OR 'aryl hydrocarbon receptor':ti,ab,kw OR lipidomic*:ti,ab,kw OR oxylipin*:ti,ab,kw OR sphingolipid*:ti,ab,kw OR 'volatile organic compound':ti,ab,kw OR 'volatile organic compounds':ti,ab,kw OR voc:ti,ab,kw OR 'microbial function':ti,ab,kw OR 'microbial functions':ti,ab,kw OR metagenom*:ti,ab,kw)<br>AND<br>('patient reported outcome'/exp OR 'quality of life'/exp OR 'fatigue'/exp OR 'depression'/exp OR 'anxiety'/exp OR 'sleep disorder'/exp OR 'pain'/exp OR 'patient reported outcome':ti,ab,kw OR 'patient reported outcomes':ti,ab,kw OR pro:ti,ab,kw OR prom:ti,ab,kw OR 'quality of life':ti,ab,kw OR hrqol:ti,ab,kw OR fatigue:ti,ab,kw OR tiredness:ti,ab,kw OR depression:ti,ab,kw OR depressive:ti,ab,kw OR anxiety:ti,ab,kw OR mood:ti,ab,kw OR sleep:ti,ab,kw OR insomnia:ti,ab,kw OR pain:ti,ab,kw OR urgency:ti,ab,kw OR 'stool frequency':ti,ab,kw OR 'symptom burden':ti,ab,kw OR 'bowel confidence':ti,ab,kw))
Scopus	11 June 2026; no date, language, age, species, or study-design filters at the database-search stage.	TITLE-ABS-KEY(("inflammatory bowel disease" OR IBD OR Crohn* OR "ulcerative colitis" OR "IBD-unclassified" OR "IBD unclassified")<br>AND<br>(metabolomic* OR metabolite* OR "metabolic signature" OR "metabolic signatures" OR "microbiota-derived metabolite" OR "microbiota-derived metabolites" OR "short-chain fatty acid" OR "short-chain fatty acids" OR SCFA OR butyrate OR propionate OR acetate OR "bile acid" OR "bile acids" OR tryptophan OR kynurenine OR serotonin OR indole* OR AhR OR "aryl hydrocarbon receptor" OR lipidomic* OR oxylipin* OR sphingolipid* OR "volatile organic compound" OR "volatile organic compounds" OR VOC OR "microbial function" OR "microbial functions" OR metagenom*)<br>AND<br>("patient-reported outcome" OR "patient-reported outcomes" OR PRO OR PROM OR "quality of life" OR HRQoL OR fatigue OR tiredness OR depression OR depressive OR anxiety OR mood OR sleep OR insomnia OR pain OR urgency OR "stool frequency" OR "symptom burden" OR "bowel confidence"))
Web of Science Core Collection	11 June 2026; no date, language, age, species, or study-design filters at the database-search stage.	TS=(("inflammatory bowel disease" OR IBD OR Crohn* OR "ulcerative colitis" OR "IBD-unclassified" OR "IBD unclassified")<br>AND<br>(metabolomic* OR metabolite* OR "metabolic signature" OR "metabolic signatures" OR "microbiota-derived metabolite" OR "microbiota-derived metabolites" OR "short-chain fatty acid" OR "short-chain fatty acids" OR SCFA OR butyrate OR propionate OR acetate OR "bile acid" OR "bile acids" OR tryptophan OR kynurenine OR serotonin OR indole* OR AhR OR "aryl hydrocarbon receptor" OR lipidomic* OR oxylipin* OR sphingolipid* OR "volatile organic compound" OR "volatile organic compounds" OR VOC OR "microbial function" OR "microbial functions" OR metagenom*)<br>AND<br>("patient-reported outcome" OR "patient-reported outcomes" OR PRO OR PROM OR "quality of life" OR HRQoL OR fatigue OR tiredness OR depression OR depressive OR anxiety OR mood OR sleep OR insomnia OR pain OR urgency OR "stool frequency" OR "symptom burden" OR "bowel confidence"))
